# An exploratory assessment of GPT-4o and GPT-4 performance on the Japanese National Dental Examination

**DOI:** 10.1016/j.sdentj.2024.11.006

**Published:** 2024-11-26

**Authors:** Masaki Morishita, Hikaru Fukuda, Shino Yamaguchi, Kosuke Muraoka, Taiji Nakamura, Masanari Hayashi, Izumi Yoshioka, Kentaro Ono, Shuji Awano

**Affiliations:** a Division of Clinical Education Development and Research, Department of Oral Function, Kyushu Dental University, Kitakyushu, Japan; b Health Information Management Office, Kyushu Dental University Hospital, Kitakyushu, Japan; c Division of Maxillofacial Surgery, Department of Physical Function, Kyushu Dental University, Kitakyushu, Japan; d School of Oral Health Sciences, Kyushu Dental University, Kitakyushu, Japan; e Division of Periodontology, Department of Oral Function, Kyushu Dental University, Kitakyushu, Japan; f Vice President, Kyushu Dental University, Kitakyushu, Japan; g Division of Oral Medicine, Department of Physical Function, Kyushu Dental University, Kitakyushu, Japan; h Division of Physiology, Department of Health Promotion, Kyushu Dental University, Kitakyushu, Japan

**Keywords:** GPT-4o, GPT-4, Japanese National Dental Examination, Education tool

## Abstract

**Background and Objectives:**

Multiple large language models (LLMs) have been released since 2022, including OpenAI’s GPT-3.5 and GPT-4. The latest model, GPT-4o, introduced on May 13, 2024, significantly improves GPT-4. Previous studies have shown the potential of LLMs as educational tools in medical and dental exams. This study evaluates the accuracy of GPT-4 and GPT-4o responses for the Japanese National Dental Examination (JNDE) to assess their potential as educational tools for dental education.

**Materials and methods:**

We obtained the dataset of the 117th JNDE, administered in January 2024, consisting of 360 questions. After excluding questions with images and inappropriate ones, 202 questions were selected. GPT-4 and GPT-4o were used to generate responses. Standardized prompts ensured consistent input. Data analysis used Qlik Sense® and GraphPad Prism, employing Fisher’s exact test.

**Results:**

GPT-4o showed a significantly higher correct response rate (73.8%) than GPT-4 (63.3%). In the compulsory section, GPT-4o achieved 88.6% accuracy, significantly higher than GPT-4′s 74.3%. Though not statistically significant, the general section saw an improvement with GPT-4o (66.4%) over GPT-4 (58.0%).

**Conclusion:**

GPT-4o significantly outperformed GPT-4 in accuracy for JNDE questions, suggesting its improved potential as an educational tool in dental education. Further studies are needed to evaluate GPT-4o’s capabilities with visual materials and in diverse question sets to fully ascertain its utility in educational settings.

## 1 Introduction

Since 2022, several large language models have been released, starting with GPT-3.5 by OpenAI, followed by Google’s Bard, Gemini, Microsoft’s Microsoft Copilot, and Anthropic’s Claude3 (OpenAI, , 2022, Google, , 2024a, Google, , 2024b, Microsoft, , 2023, Anthropic, , 2024). OpenAI has continued introducing new ChatGPT models, including GPT-4, GPT-4 V(ision) with image recognition capabilities, and the latest GPT-4o(mni) released on May 13, 2024 (OpenAI, , 2024a, OpenAI, , 2024b, OpenAI, , 2024c). Many reports have utilized various LLMs to assess the accuracy of responses to questions from national examinations for medical doctors and health care professionals like dentists, nurses, and pharmacists, demonstrating that LLMs have the potential to serve as valuable educational support tools (Yanagita et al., 2023, Takagi et al., 2023, Taira et al., 2023, Kunitsu, 2023, Sato and Ogasawara, 2024, Kaneda et al., 2023, Doi et al., 2024, Sawamura et al., 2024, Kohiyama et al., 2024, Kobayashi, 2023). In our prior analyses, we assessed the accuracy of responses to questions from the Japanese national examination for dental hygienists by employing multiple LLMs (Yamaguchi et al., 2024). Additionally, we compared the accuracy of responses to questions from the Japanese national dental examination using GPT-3.5 and GPT-4 (Morishita et al., 2024). Some reports utilized the GPT-4 V with image recognition capabilities to evaluate responses to questions containing qualification material such as x-ray and photographs, indicating the potential applications of GPT-4 and GPT-4 V as educational support tools in medical and dental education (Morishita et al., 2024, Morishita et al., 2023, Nakao et al., 2024, Takagi et al., 2024). According to OpenAI, the GPT-4o has significantly improved over the GPT-4 (OpenAI, 2024c).

Therefore, this study aims to evaluate the accuracy of responses generated by the Japanese National Dental Examination using the GPT-4 and the latest GPT-4o model to determine its potential as an educational tool for dental education.

## 2 Materials and methods

### 2.1 Obtaining and processing data from the Japanese National dental Examination

We downloaded the questions dataset of the 117th Japanese National Dental Examination (JNDE), administered in January 2024, from Japan's Ministry of Health, Labour and Welfare website (The Ministry of Health, Labour and Welfare in Japan, 2024a). The JNDE consists of 360 questions, and in the present study, only questions that did not include figures or images were extracted; 208 questions were selected. The Ministry of Health, Labour and Welfare in Japan evaluates examination questions after they are administered and publishes the questions eliminated from scoring as inappropriate on the web (The Ministry of Health, Labour and Welfare in Japan, 2024b). Six of the 208 questions were excluded as inappropriate; 202 were used in this study. The correct answers are the official ones published on Japan's Ministry of Health, Labor, and Welfare website. The JNDE consists mainly of multiple-choice questions with five choices: choose one correct, choose two, choose three, choose four, and choose all.

The Guidelines for the JNDE define the required essential topics, general dentistry, and each topic of dentistry (The Ministry of Health, Labour and Welfare in Japan, 2024c). The compulsory questions cover the fundamental knowledge and skills required to practice as a dentist. The general dentistry and specific dentistry sections build upon this foundational knowledge and are referred to as the general and clinical practical sections (The Ministry of Health, Labour and Welfare in Japan, 2024c). The JNDE is divided into three areas: compulsory questions, general questions, and clinical practical questions. The subspecialty of the questions was determined based on the 117th National Dental Examination question book published by Azabu Dental Academy (Azabu Dental Academy, 2024).

### 2.2 ChatGPT models

GPT-4 and GPT-4o, developed by OpenAI, were used as LLMs (OpenAI, , 2024a, OpenAI, , 2024c). Standardized prompt inputs and question texts were entered into LLM's web interface, and all responses obtained were recorded. The input was performed on 14 May 2024. All standardized prompt inputs and question texts were entered in Japanese. A prompt is “an instruction given to an LLM to enforce a rule, automate a process, or guarantee a specific quality and quantity of the generated output” (White et al., 2023).

We standardized the format of the prompts when entering the question text as follows: “You are a student for the national dental examination. Read the question text and output only the correct answer choices a, b, c, d, and e, according to the question text.”.

### 2.3 Data and statistical analysis

We used Qlik Sense® Enterprise August 2022 Patch 2 (Qlik Technologies Inc., King of Prussia, PA, USA) for data analysis. We used GraphPad Prism 9.5.1 (GraphPad Software, Boston, MA, USA) for statistical analysis employing Fisher's exact test.

## 3 Results

Table 1 shows the percentage of correct answers for the compulsory, general questions, and clinical practical questions using GPT-4 and 4o. In the compulsory section, GPT-4o showed a statistically significant higher percentage of correct responses at 88.6 %, compared to 74.3 % for the GPT-4. The correct response rate was also higher for GPT-4o in the general section, with 66.4 % compared to 58.0 % for GPT-4, but this difference was not statistically significant. Overall, GPT-4o demonstrated statistically significantly higher percentages of correct responses at 73.8 %, compared to 63.3 % for GPT-4.

**Table 1:** Performance of GPT-4 and 4o in the Japanese National Dental Examination.

	GPT	Number of questions (n)	Correct answers (n)	Incorrect answers (n)	Correct answers (%)	P-value
Compulsory	4	70	52	18	74.3	0.0491
	4o	70	62	8	88.6	
General	4	131	76	55	58.0	0.2025
	4o	131	87	44	66.4	
Clinical practical	4	1	0	1	0.0	>0.9999
	4o	1	0	1	0.0	
Total	4	202	128	74	63.3	0.0319
	4o	202	149	53	73.8	

Table 2 shows the percentage of correct answers for each dental subject in the compulsory section, arranged by the number of questions. There was no statistically significant difference in the percentage of correct answers between GPT-4 and GPT-4o across all dental subjects.

**Table 2:** Comparison of the percentage of correct answers between GPT-4 and GPT-4o by dental subject in the compulsory section.

	GPT	Number of questions (n)	Correct answers (n)	Incorrect answers (n)	Correct answers (%)
Oral public health	4	16	13	3	81.3
	4o	16	15	1	93.8
Pediatric dentistry	4	8	4	4	50.0
	4o	8	5	3	62.5
Oral surgery	4	7	6	1	85.7
	4o	7	7	0	100.0
Oral pharmacology	4	6	6	0	100.0
	4o	6	6	0	100.0
Orthodontics	4	5	1	4	20.0
	4o	5	3	2	60.0
Oral biochemistry	4	4	4	0	100.0
	4o	4	4	0	100.0
Oral microbiology	4	4	4	0	100.0
	4o	4	4	0	100.0
Periodontics	4	3	2	1	66.7
	4o	3	3	0	100.0
Oral physiology	4	3	3	0	100.0
	4o	3	3	0	100.0
Oral pathology	4	3	3	0	100.0
	4o	3	3	0	100.0
Dental anesthesiology	4	3	3	0	100.0
	4o	3	3	0	100.0
Crown and bridge	4	2	1	1	50.0
	4o	2	1	1	50.0
Endodontics	4	2	1	1	50.0
	4o	2	2	0	100.0
Full denture	4	2	1	1	50.0
	4o	2	1	1	50.0
Restorative dentistry	4	1	0	1	0.0
	4o	1	1	0	100.0
Oral radiology	4	1	0	1	0.0
	4o	1	1	0	100.0

Table 3 shows the percentage of correct answers for each dental subject in the general section, arranged by the number of questions. There was no statistically significant difference in the percentage of correct answers between GPT-4 and GPT-4o across all dental subjects.

**Table 3:** Comparison of the percentage of correct answers between GPT-4 and GPT-4o by dental subject in the general section.

	GPT	Number of questions (n)	Correct answers (n)	Incorrect answers (n)	Correct answers (%)
Oral public health	4	25	18	7	72.0
	4o	25	17	8	68.0
Dental anesthesiology	4	16	13	3	81.3
	4o	16	14	2	87.5
Oral surgery	4	14	10	4	71.4
	4o	14	11	3	78.6
Pediatric dentistry	4	9	5	4	55.6
	4o	9	7	2	77.8
Crown and bridge	4	7	2	5	28.6
	4o	7	3	4	42.9
Orthodontics	4	7	1	6	14.3
	4o	7	3	4	42.9
Dental anatomy	4	6	3	3	50.0
	4o	6	2	4	33.3
Periodontics	4	6	3	3	50.0
	4o	6	4	2	66.7
Oral physiology	4	6	4	2	66.7
	4o	6	5	1	83.3
Oral pathology	4	6	3	3	50.0
	4o	6	4	2	66.7
Endodontics	4	5	2	3	40.0
	4o	5	2	3	40.0
Oral microbiology	4	5	3	2	60.0
	4o	5	3	2	60.0
Oral radiology	4	4	1	3	25.0
	4o	4	3	1	75.0
Oral pharmacology	4	4	3	1	75.0
	4o	4	4	0	100.0
Restorative dentistry	4	3	1	2	33.3
	4o	3	1	2	33.3
Partial denture	4	3	3	0	100.0
	4o	3	2	1	66.7
Full denture	4	2	0	2	0.0
	4o	2	0	2	0.0
Dental materials	4	2	0	2	0.0
	4o	2	1	1	50.0
Oral biochemistry	4	1	1	0	100.0
	4o	1	1	0	100.0

Table 4 shows the percentage of correct answers and other relevant information based on the number of correct answers provided in the question text. When there was only one correct answer, GPT-4o demonstrated a statistically significantly higher percentage of correct answers than GPT-4, 83.3 % and 71.1 %, respectively. In cases with two correct responses, GPT-4o and GPT-4 achieved 64.3 % and 53.6 % respectively. GPT-4o outperformed GPT-4 regarding correct responses, although this difference was not statistically significant. When the correct answers numbered three or four, the correct response rates for GPT-4 and GPT-4o were identical. For 'choose all' questions, the correct response rate was 50 % for GPT-4o and 0 % for GPT-4. There was an improvement for GPT-4o, but this was not statistically significant.

**Table 4:** Comparison of GPT-4 and GPT-4o based on the number of questions, correct answers, incorrect answers, and percentage of correct answers to the number of correct answers specified in the question text.

Number of specified correct answers	GPT	Number of questions (n)	Correct answers (n)	Incorrect answers (n)	Correct answers (%)	P-value
1	4	114	81	33	71.1	0.0396
	4o	114	95	19	83.3	
2	4	56	30	26	53.6	0.3369
	4o	56	36	20	64.3	
3	4	26	15	11	57.7	>0.9999
	4o	26	15	11	57.7	
4	4	4	2	2	50.0	>0.9999
	4o	4	2	2	50.0	
all	4	2	0	2	0.0	>0.9999
	4o	2	1	1	50.0	

## 4 Discussion

In this study, we compared the accuracy of responses for the JNDE question dataset between GPT-4 and the latest model, GPT-4o. The results indicated that GPT-4o had significantly higher accuracy rates than GPT-4 across the 202 questions in our dataset. OpenAI demonstrated that GPT-4o achieved GPT-4 Turbo performance levels in text, reasoning, and coding intelligence, as measured by traditional benchmarks, and set new high standards in multilingual and audio capabilities (OpenAI, 2024c). The OpenAI text evaluation also revealed that GPT-4o and GPT-4 achieved 88.7 % and 86.4 % accuracy in Massive Multitask Language Understanding, respectively (OpenAI, 2024c).

A report compared 22 LLMs using multi-image and long-context methods and found that GPT-4o achieved the highest score (Song et al., 2024). Despite the differences in datasets used in their study and ours, GPT-4o remains an outstanding model. The JNDE's passing criterion for the compulsory section is 80 % correct on absolute assessment. This criterion could not be exceeded by 74.3 % in GPT-4 but was exceeded by 88.6 % in GPT-4o (Table 1).

Table 2, Table 3 show no statistically significant difference for all subjects between GPT-4 and GPT-4o in both the compulsory and general sections. The dental subjects achieved different percentages of correct answers in the general section compared to the compulsory section. This difference can be attributed to the increased difficulty of the questions in the general section compared to the compulsory section (Morishita et al., 2024).

Table 4 shows that when the question text specified one correct answer, the GPT-4o was statistically significantly better than the GPT-4. However, when the question required 2, 3, or 4 correct answers, GPT-4 and GPT-4o could not have provided a better response. This suggests that having to choose from a higher number of correct answers may still be a weakness for the GPT. Even with GPT-4o, it is challenging for AI to select all the answers, and still challenging for humans (Toyama and Nakamura, 2014).

Previous reports evaluating GPT-4 by inputting Japanese question text have also shown that GPT-4 performance is significantly improved over GPT-3.5, allowing GPT-4 to pass the Japanese medical and healthcare professional national examinations without exceptional prompt engineering (Yanagita et al., 2023, Takagi et al., 2023, Taira et al., 2023, Kunitsu, 2023, Sato and Ogasawara, 2024, Kaneda et al., 2023, Doi et al., 2024, Sawamura et al., 2024, Kohiyama et al., 2024, Kobayashi, 2023), indicating that the recognition accuracy of languages other than English has also improved, and we believe that the effect of inputting question text into the web interface in Japanese on the correct response rate is minimal.

One limitation of our study is that we could not evaluate the GPT-4o's multimodality capabilities, especially its supposedly improved image recognition abilities, as issues involving visual materials such as X-rays and intraoral photographs were excluded. Therefore, a future assessment of GPT-4o's functionality is necessary, focusing on problems related to visual materials. Additionally, the potential for bias in the study results must be addressed due to the limited number of questions per dental subject. The accuracy of responses to GPT-4 and GPT-4o was assessed using only 117 JNDE questions. It is essential to compare with multiple JNDE question datasets in the future.

It is important to note that this study's results were based on the publicly available models at the time of the study, and caution should be exercised when interpreting the results. A further limitation of this study is that studies similar to this study have yet to be reported using GPT-4o, released on 13 May 2024, and could not be discussed in depth.

The study examined the accuracy of GPT-4 and GPT-4o responses in JNDE. The high rate of correct responses from GPT-4o in certain areas suggests its potential effectiveness as an educational support tool. However, further research is needed to identify specific areas where the model may need improvement or where additional aids and teacher guidance might be beneficial. GPT-4o is also anticipated to be a tool for promoting self-study by targeting students' weak areas. For example, GPT-4o could generate numerous questions in these areas, allowing students to study them repeatedly. Further data collection and research are necessary to consider integrating GPT-4o and other LLMs into the educational curriculum.

## 5 Conclusion

In conclusion, there was a statistically significant increase in the percentage of JNDEs answered correctly by the GPT4o compared to the GPT-4 for questions that did not include images or diagrams, suggesting that the potential value of the GPT-4o as a support tool in dental education has increased further than the GPT-4. Furthermore, improvements to the GPT model may continue to increase its value as an educational support tool.

## Funding source

This research did not receive any specific grant from funding agencies in the public, commercial, or not-for-profit sectors.

## Declaration of Competing Interest

The authors declare that they have no known competing financial interests or personal relationships that could have appeared to influence the work reported in this paper.
